# Differential Expression of Vitreous Proteins in Young and Mature New Zealand White Rabbits

**DOI:** 10.1371/journal.pone.0153560

**Published:** 2016-04-18

**Authors:** Ying Liu, Rachida A. Bouhenni, Craig P. Dufresne, Richard D. Semba, Deepak P. Edward

**Affiliations:** 1 Wilmer Eye Institute, Johns Hopkins University School of Medicine, Baltimore, United States of America; 2 King Khaled Eye Specialist Hospital, Riyadh, Saudi Arabia; 3 Summa Health System, Akron, Ohio, United States of America; 4 Thermo Fisher Scientific, West Palm Beach, Florida, United States of America; 5 Changsha Aier Eye Hospital, Changsha, China; Tsinghua University, CHINA

## Abstract

Different anatomical regions have been defined in the vitreous humor including central vitreous, basal vitreous, vitreous cortex, vitreoretinal interface and zonule. In this study we sought to characterize changes in the proteome of vitreous humor (VH) related to compartments or age in New Zealand white rabbits (NZW). Vitreous humor was cryo-collected from young and mature New Zealand white rabbit eyes, and dissected into anterior and posterior compartments. All samples were divided into 4 groups: Young Anterior (YA), Young Posterior (YP), Mature Anterior (MA) and Mature Posterior (MP) vitreous. Tryptic digests of total proteins were analyzed by liquid chromatography followed by tandem mass spectrometry. Spectral count was used to determine the relative protein abundances and identify proteins with statistical differences between compartment and age groups. Western blotting was performed to validate some of the differentially expressed proteins. Our results showed that 231, 375, 273 and 353 proteins were identified in the YA, YP, MA and MP respectively. Fifteen proteins were significantly differentially expressed between YA and YP, and 11 between MA and MP. Carbonic anhydrase III, lambda crystallin, alpha crystallin A and B, beta crystallin B1 and B2 were more abundant in the anterior region, whereas vimentin was less abundant in the anterior region. For comparisons between age groups, 4 proteins were differentially expressed in both YA relative to MA and YP relative to MP. Western blotting confirmed the differential expression of carbonic anhydrase III, alpha crystallin B and beta crystallin B2. The protein profiles of the vitreous humor showed age- and compartment-related differences. This differential protein profile provides a baseline for understanding the vitreous compartmentalization in the rabbit and suggests that further studies profiling proteins in different compartments of the vitreous in other species may be warranted.

## Introduction

The vitreous humor (VH) is a transparent gel-like extracellular matrix that occupies the cavity between the lens and the retina. Different anatomical regions have been defined including central vitreous, basal vitreous, vitreous cortex, vitreoretinal interface and zonule[1]. In addition to its physical functions, the VH also contains many proteins accumulated by local secretion, filtration from the blood, or diffusion from the surrounding tissues and vasculature[2–4]. These proteins may alter the physiochemical properties of this matrix and affect processes occurring in the structures in contact with or adjacent to the VH[5]. Identification and quantitation of vitreous proteins could reveal the disease state, provide additional information about disease mechanisms and improve our understanding of the pathogenesis of some eye diseases including vitreoretinal diseases and intraocular inflammation[3].

Different compartments of VH exist that include the vitreous base, core, cortex, and anterior hyaloid[6]. These various compartments are either in contact with the lens/ciliary body anteriorly, or with the retinal surface posteriorly. Vitreous proteins may originate from the retina, ciliary body, lens, retinal pigmented epithelium, or the systemic circulation[6, 7]. The physiological and pathological conditions of the lens/ciliary body or the retina may affect the protein composition of the VH. Therefore, the macromolecular composition of VH may vary by the anatomical region where the sample is acquired. Skeie and Mahajan studied different compartments of the vitreous, and analyzed their protein content by one-dimensional (1D) sodium dodecyl sulphate-polyacrylamide gel electrophoresis (SDS-PAGE)[6]. The authors suggested that there are differentially expressed proteins in the various vitreous body substructures. However, these proteins were not identified. Identification of specific proteins may provide greater insight into the clinically identified vitreous compartments and reveal candidate molecules underlying various vitreoretinal or intraocular inflammatory diseases.

The protein profiles of VH may also vary by the age of the subject/patient, the state of the lens, and the presence of any vitreous pathology[1, 2, 7, 8]. Specifically, vitreous changes with age lead to a dynamic change in vitreous compartments as the vitreous liquefies and vitreous channels and compartments collapse, thus potentially affecting diffusion of intravitreally injected drugs to the posterior retina[9]. Thus, the characterization of age related changes in the vitreous compartments in healthy animals may provide baseline information that might help us understand pathologic alterations in the vitreous, develop drugs or drug-delivery techniques that overcome barriers to drug perfusion to the retina[10].

Although experimental vision research is traditionally performed on rodent models, the rabbit is still useful in modeling some common diseases such as glaucoma, age-related macular degeneration, light-induced retinopathies, cataract and uveitis since rabbits can be easily handled and share more common anatomical and biochemical features with humans compared to rodents. These include longer life spans and larger eye size which provide a larger VH volume[11]. In addition, the rabbit is a particularly useful animal model in studying intravitreal pharmacokinetics[12]. It is therefore necessary and meaningful to perform studies on the protein complexity of the rabbit vitreous. In this study, using liquid chromatography-tandem mass spectrometry (LC-MS/MS), we aimed to investigate differences in protein profiles between the two anatomical regions (anterior and posterior) of the vitreous body in rabbits at two different ages referred to as young and mature. This study intended to provide a basic understanding of vitreous compartmentalization and to help identify future biomarkers for various vitreoretinal diseases with respect to age and specific location in the vitreous humor.

## Materials and Methods

### Tissue procurement

Two sets of freshly enucleated eyes of New Zealand white (NZW) (n = 8/set), young (8 weeks old, n = 4), and mature (6 months and older, n = 4; rabbits reach sexual maturity at 6 month [13]) rabbits were obtained from Pel-freeze Biologicals (n = 8, Rogers, AR) and Johns Hopkins University (n = 8, JHU) within 10 min of sacrifice with euthanasia using pentobarbital containing solution (80–100 mg/kg, Fatal plus, Butler Schein Animal Health, Dublin, OH) intravenously under a different non ocular protocol approved by the Institutional Animal Care and Use Committee at JHU. Since these eyes were obtained after euthanasia under a different protocol, animal welfare clearance was not required. The first set was processed for sample preparation for proteomics and the second for western blotting. Upon arrival, the eyes were snap-frozen and stored at -80°C. The frozen eyes were then bisected in the pupil-optic nerve axis. Under a dissecting microscope, the frozen vitreous posterior to the anterior zonular attachments to the lens and anterior to the vitreous base (the vitreous between the ciliary body and the lens) was dissected using a No #11 ophthalmic blade and labeled as anterior vitreous compartment; the vitreous adjacent to the posterior retina (2–3 mm to retina) was collected and labeled as posterior vitreous compartment. Care was taken not to contaminate the vitreous sample with the adjacent cellular structures. The vitreous was thawed on ice and centrifuged at 4°C at 2,000 x g for 10 minutes. Protease inhibitor cocktail (Roche Applied Science, IN) was added to the samples which were stored at -80°C until further processing.

### Liquid chromatography/tandem mass spectrometry (LC-MS/MS)

#### Sample preparation

VH samples were processed individually as described previously[14, 15]. Briefly, 100 μl of VH was mixed with 100 μl of acrylamide/bis (30%T/2.67%C), 10 μl of 10% ammonium persulfate and 5 μl of TEMED in the lid of a microcentrifuge tube to form a gel and then transferred into the tube. Following fixation in 1 ml of 40% methanol and 7% acetic acid for 30 minutes, the gel pieces were washed twice with water, twice with 50% acetonitrile, once with 50mM ammonium bicarbonate/50% acetonitrile, and once with 100 mM ammonium bicarbonate/50% acetonitrile for 30 minutes each. Samples were subsequently dried in SpeedVac. Then 200 μl of 100 mM ammonium bicarbonate containing 1.0 μg trypsin (Promega Corporation, Madison, WI) was added to each gel piece and incubated overnight at 37°C. Tryptic peptides were extracted with 70% acetonitrile/0.1% formic acid and dried. Peptides were dissolved in 6M guanidine-HCl in 25mM potassium phosphate buffer with 5mM DTT. Peptide clean-up/desalting was performed using C_18_ ZipTip (Millipore, Billerica, MA) columns. Peptides bound to C_18_ were washed 5 times with water/0.1% formic acid and eluted with 70% Acetonitrile/0.1% formic acid into chromtech glass inserts, and dried.

#### LC-MS/MS spectrometry

LC-MS/MS was performed using a one-hour gradient of 2–30% acetonitrile with 0.1% formic acid using an EASY-Spray source coupled with an Orbitrap Elite hybrid mass spectrometer (Thermo Scientific). EASY-Spray source was run at 35°C using a 25cm x 75μm integrated spray tip column spraying at 350 nanoliters/minute. Peptides were trapped at 980 bar on a 2cm x 75μm trapping column (Thermo Scientific). The trap was a 3μm particle, and the column was 2μm Acclaim PepMap C_18_.

#### Data Analysis

MS raw data was batch processed using i3D (Shimadzu and Integrated Analysis), X!Tandem and OMSSA search engines, and the UniProt sequence database. The following parameters were used: trypsin was chosen for protein digestion; carbamidomethylation was set as fixed modification and oxidation was set as variable modification. The missed cleavages was 2, peptide mass tolerance was 10 ppm and the MS/ MS fragment tolerance was limited to 0.5 Da. Scaffold 4.4.3 (2015, Proteome Software) was used for result validation and quantitative spectral counting value of each protein was normalized by the values of total proteins. As the rabbit genome is incomplete, we used the Uniprot protein database search with mammalian taxonomy for uncharacterized protein identifications. Those uncharacterized proteins were replaced by reviewed proteins from other similar species with high identification scores. In case of multiple protein names, only one protein name was used based on homology. Spectral count was used to determine the relative abundance of proteins in each sample as previously described[16]. Probability score was filtered at 90%. Positive protein identification was based on at least 2 unique matched peptides. Only those proteins detected in at least 2 out of 4 samples in any group were used for statistical analysis. G test (log likelihood ratio test for goodness of fit) was then used to determine the significant differences in proteins abundance between groups as previously described[17]. The p-values were adjusted with the Holm-Sidak method of correction for multiple comparisons. Proteins with an adjusted P-value < 0.05 were filtered to identify those differentially abundant proteins in one group relative to the other group.

### Western blot analysis

To validate the expression of specific proteins, western blotting (WB) was performed. Protein concentration was determined using the Bradford assay (Biorad laboratories, Hercules, CA). Equal amounts (20μg) of sample were loaded into a 4–15% SDS PAGE (BioRad Laboratories, Hercules, CA). The proteins were then transferred into nitrocellulose membranes (Life Technologies, Carlsbad, CA). Membranes were blocked with 5% bovine serum albumin (BSA) (w/v) for 1 h at room temperature and incubated overnight at 4°C with the appropriate primary antibody: mouse anti-alpha B crystallin (CRYAB, 1:400, Lifespan Biosciences, Seattle, WA), mouse anti-beta crystallin (CRYBB2, 1:3000, Abcam, Cambridge, MA), rabbit anti-carbonic anhydrase III (CA3, 1:1500, Abcam, Cambridge, MA), mouse anti-vimentin (VIM, 1:2000, Biogenex, Fremont, CA) followed by incubation with horseradish-peroxidase (HRP)-conjugated goat anti-mouse secondary antibody (1:5000; Sigma-Aldrich, St.Louis, MO) or goat anti-rabbit secondary antibody (1:2000; Jackson ImmunoResearch Laboratories, West Grove, PA) for 1 hour at room temperature. Signal was detected by enhanced chemiluminescence using SuperSignal West Pico kit (Thermo Scientific, Rockford, IL). Densitometry was performed using Image J (NIH, Bethesda, MD) to compare between groups.

### Statistical analysis

G test followed by post hoc Holm-Sidak test was performed to determine the proteins with significantly different levels between groups as described above, while Mann Whitney U test was used to determine the significant differences in protein abundance when western blots were analyzed by densitometry. P < 0.05 was considered statistically significant.

## Results

A total of 16 independent vitreous samples were divided into 4 groups according to age and location of vitreous: anterior and posterior vitreous compartments from young rabbits (briefly young anterior and posterior vitreous respectively); anterior and posterior vitreous compartments from mature rabbits (briefly mature anterior and posterior vitreous respectively).

### Protein identification

**Fig 1:**
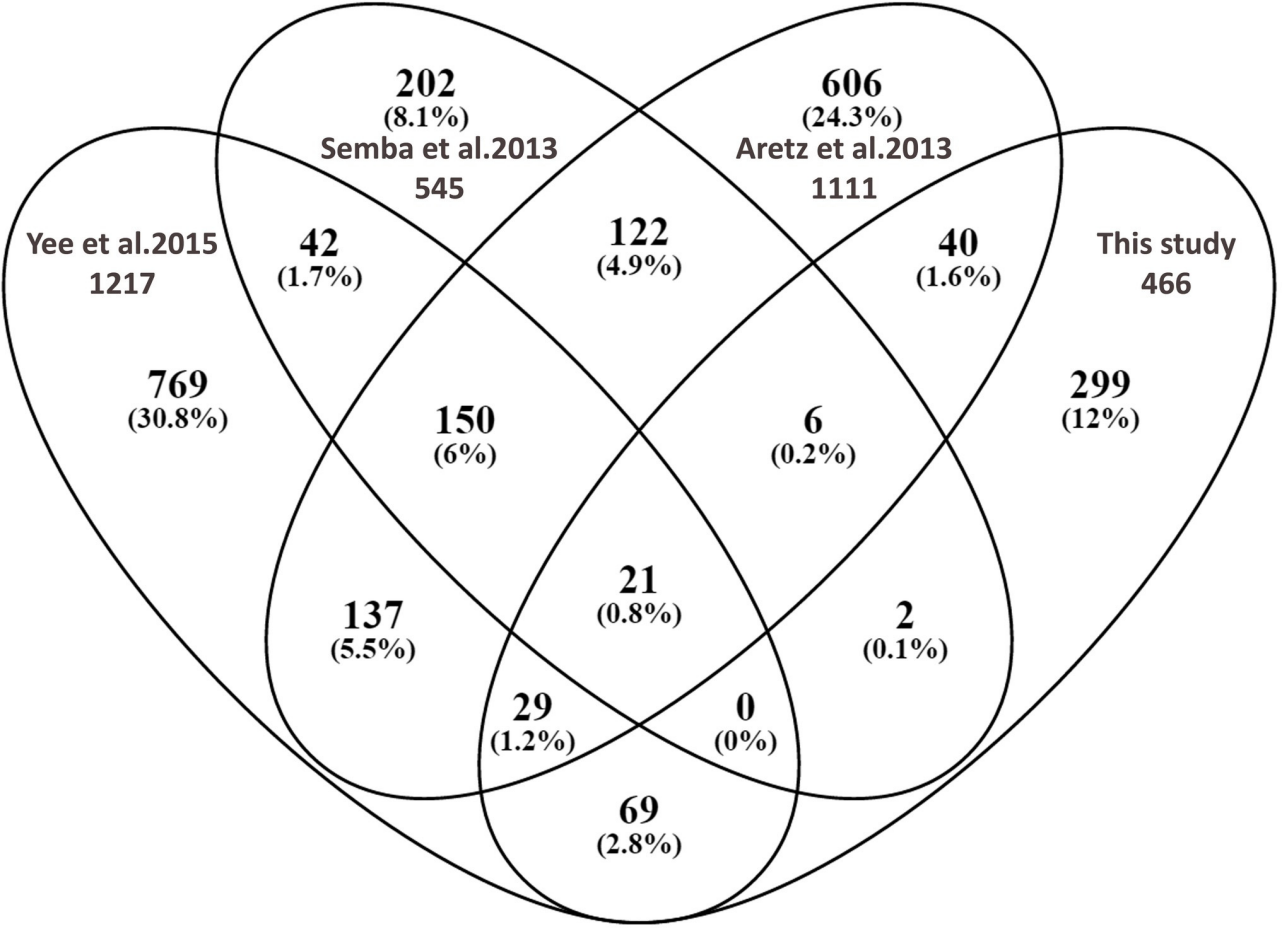
Venn diagram comparing the proteomes of four human vitreous studies (present study versus Yee et al. versus Aretz et al. Versus versus Semba et al. (made with online venn diagram plotter at http://bioinfogp.cnb.csic.es/tools/venny).

The total number of non-redundant proteins identified by LC-MS/MS in our study was 466, based on the criteria that only proteins identified with at least 2 peptides in at least 2 samples of one individual group were selected with identity confidence of 90% and higher. A previous review performed by Semba et al. reported a total of 545 non-redundant proteins in human VH [18]. Other studies such as those performed by Aretz et al.[19] and Murphy et al.[20] identified 1111 and 1205 proteins from human vitreous respectively, the latter included proteins identified with 1 unique peptide. Recently Yee et al detected 1217 proteins in fetal and young adult human vitreous, and identified differences between embryonic and young adult vitreous proteomes[21]. In this study, we identified only 466 proteins, 299 of these are new and have not been previously reported in either of aforementioned studies excluding that by Murphy et al[20] (Fig 1). Full lists of all identified proteins and newly identified proteins in our study were provided in the supplemental tables (S1 Table).

**Fig 2:**
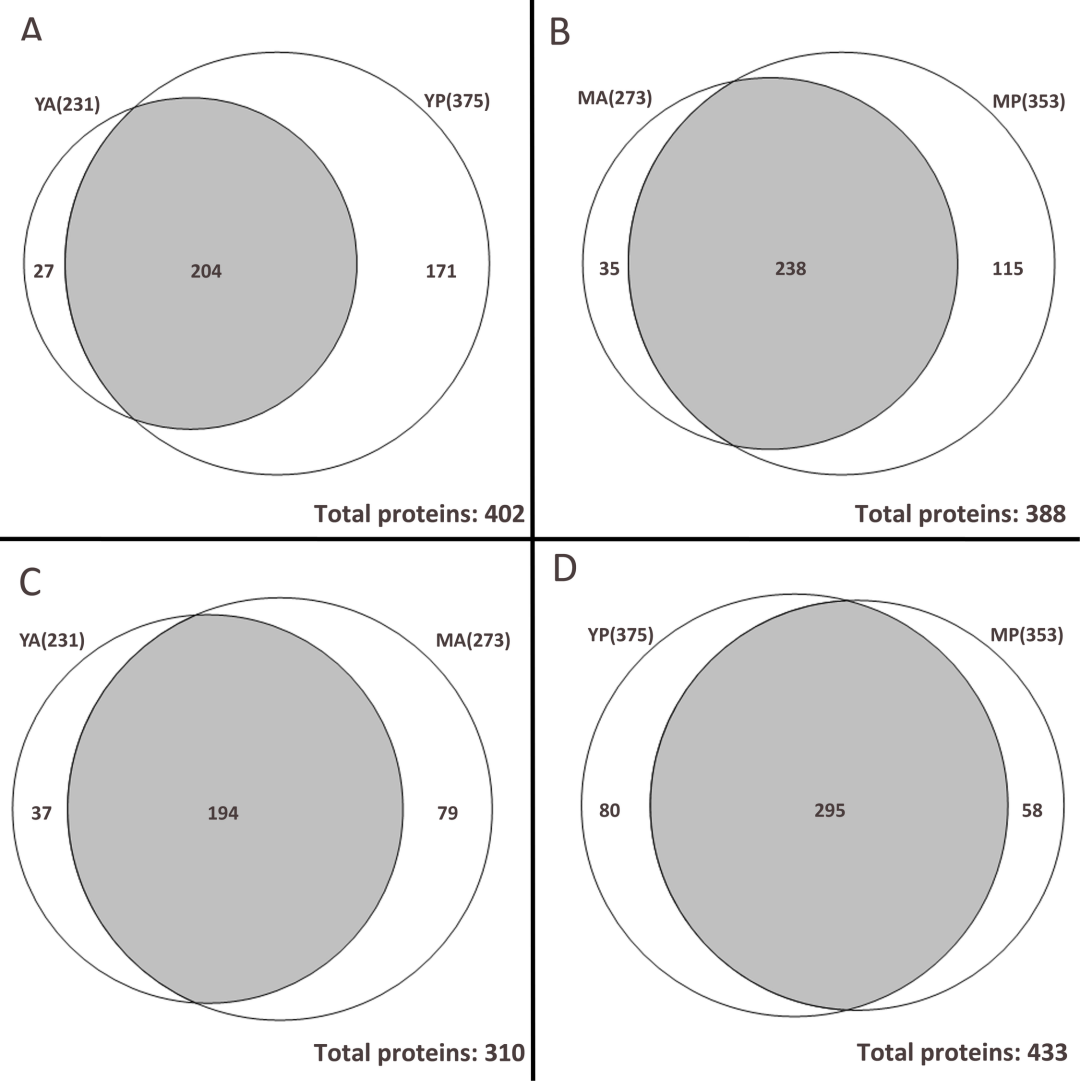
Venn diagram of proteins identified in anterior or posterior vitreous of young and mature rabbits by LC-MS/MS. (A) 231 proteins were identified in the anterior vitreous of young rabbit (YA) while 375 proteins were identified in the posterior vitreous of young rabbit (YP), among them were 204 proteins shared by these two groups; (B) The total numbers of proteins detected in the anterior and posterior vitreous of mature rabbit (MA and MP respectively) were 273 and 353, they shared 238 proteins; (C) The number of common proteins shared by YA and MA was 194; (D) 295 proteins were identified in both YP and MP.

Furthermore, as shown in Fig 2A, a total of 402 proteins were identified in young rabbits. Among them, 231 proteins were identified in young anterior vitreous while 375 proteins in young posterior vitreous. These two groups shared 204 proteins. Similarly, a total of 388 proteins were identified in the mature rabbits (Fig 2B). Among these, 273 and 353 proteins were identified in mature anterior and mature posterior vitreous, respectively. These two groups shared 238 proteins. When samples were grouped by region, a total of 310 proteins were identified in anterior compartment and 194 proteins were found common to young anterior and mature anterior vitreous (Fig 2C). In the posterior compartment, 433 proteins were identified and 295 proteins were common to young posterior and mature posterior vitreous (Fig 2D).

Complete lists of identified proteins in each group were provided in supplemental data (S2 Table). These proteins were then further analyzed for comparison between groups at same age or region as follows:

#### Young anterior vitreous versus young posterior vitreous

In young rabbits, 402 proteins were compared for abundance between anterior and posterior vitreous, and 15 proteins were found differentially expressed in young anterior relative to young posterior group (Table 1 “YA VS. YP”). Eleven proteins were more abundant in the anterior vitreous, including tubulin beta chain (TUBB, p<0.001), carbonic anhydrase 3 (CA3, p<0.001), gamma-crystallin C (CRYGC, p<0.01), beta A1-crystallin (CRYBA1, p<0.001), lambda-crystallin (CRYL1, p<0.001), beta-crystallin B1 (CRYBB1, p<0.001), alpha-crystallin B chain (CRYAB, p<0.001), retinal dehydrogenase 1 (ALDH1A1, p<0.01), beta-crystallin B3 (CRYBB3, p<0.01), beta-crystallin B2 (CRYBB2, p<0.05), alpha-crystallin A chain (CRYAA, p<0.01). While vimentin (VIM, p<0.001), glutathione S-transferase Mu 1 (GSTM, p<0.001), dihydropyrimidinase-related protein 4 (DPYSL4, p<0.01) and tubulin beta-2A chain (TUBB2A, p<0.001) were found to be less abundant in the anterior compartment. S1A Fig showed the fold changes of these differentially expressed proteins between young anterior and posterior vitreous based on their normalized log ratios. Furthermore, TUBB was not identified in any samples of young posterior vitreous while DPYSL4 and TUBB2A were not identified in any samples of young anterior vitreous.

**Table 1:** Differentially expressed proteins between groups determined by G test and Holm-Sidak test.

Groups	Uniprot ID	Protein Description	Samples with positive detection	Total SC †	Total Peptides	samples with positive detection	Total SC	Total Peptides	Normalized Log ratio	Adjusted P value
YA VS. YP			YA (n = 4)			YP(n = 4)				
	P07437	Tubulin beta chain (TUBB) *	4	151	119	0	0	0	7.74	
	P16015	Carbonic anhydrase 3 (CA3)	4	39	29	2	13	10	2.01	< .001
	P02529	Gamma-crystallin C (CRYGC)	3	37	33	2	15	18	1.71	< .01
	Q95KK5	Beta A1-crystallin (CRYBA1)	4	75	56	4	38	31	1.46	< .001
	P14755	Lambda-crystallin (CRYL1)	4	158	97	4	86	62	1.36	< .001
	Q007T1	Beta-crystallin B1 (CRYBB1)	4	91	70	4	53	41	1.26	< .001
	P41316	Alpha-crystallin B chain (CRYAB)	4	107	60	4	64	46	1.23	< .001
	Q8MI17	Retinal dehydrogenase 1 (ALDH1A1)	4	63	59	4	40	38	1.14	< .01
	Q9JJU9	Beta-crystallin B3 (CRYBB3)	4	69	51	4	47	44	1.04	< .01
	A2IBH5	Beta-crystallin B2 (CRYBB2)	4	60	54	4	41	36	1.03	< .05
	P02493	Alpha-crystallin A chain (CRYAA)	4	111	60	4	84	61	0.89	< .01
	P08670	Vimentin (VIM)	4	188	131	4	407	247	-0.62	< .001
	P46409	Glutathione S-transferase Mu 1	2	6	7	4	34	31	-1.90	< .001
	O14531	Dihydropyrimidinase-related protein 4 (DPYSL4) *	0	0	0	2	20	22	-3.90	
	Q13885	Tubulin beta-2A chain (TUBB2A) *	0	0	0	2	133	92	-6.57	
MA VS. MP			MA(n = 4)			MP (n = 4)				
	P29751	Actin, cytoplasmic 1 (ACTB) *	3	51	50	0	0	0	5.79	
	Q71V39	Elongation factor 1-alpha 2 (EEF1A2) *	2	15	14	0	0	0	4.08	
	P16015	Carbonic anhydrase 3 (CA3)	4	37	30	1	4	3	3.01	< .001
	P63104	14-3-3 protein zeta/delta (YWHAZ)	2	37	30	1	5	7	2.67	< .001
	P14755	Lambda-crystallin (CRYL1)	3	184	84	2	49	33	1.97	< .001
	P41316	Alpha-crystallin B chain (CRYAB)	4	136	74	3	52	34	1.45	< .001
	P02493	Alpha-crystallin A chain (CRYAA)	4	177	63	4	73	42	1.36	< .001
	A2IBH5	Beta-crystallin B2 (CRYBB2)	4	91	68	4	43	37	1.15	< .01
	Q007T1	Beta-crystallin B1 (CRYBB1)	4	93	63	3	50	38	0.96	< .05
	P08670	Vimentin (VIM)	4	181	126	4	397	272	-1.04	< .001
	P07437	Tubulin beta chain (TUBB)	2	63	51	3	168	125	-1.31	< .001
YA VS. MA			YA(n = 4)			MA(n = 4)				
	P07437	Tubulin beta chain (TUBB)	4	151	119	2	63	51	1.45	< .001
	P63104	14-3-3 protein zeta/delta (YWHAZ)	1	3	5	2	37	30	-2.96	< .001
	G1T465	3alpha(17beta)-hydroxysteroid dehydrogenase (PGER6) *	0	0	0	2	17	18	-3.97	
	P13637	Sodium/potassium-transporting ATPase subunit alpha-3 (ATP1A3) *	0	0	0	3	19	19	-4.11	
YP VS. MP			YP (n = 4)			MP (n = 4)				
	Q13885	Tubulin beta-2A chain (TUBB2A) *	2	133	92	0	0	0	6.87	
	P29751	Actin, cytoplasmic 1 (ACTB) *	2	38	37	0	0	0	5.08	
	O14531	Dihydropyrimidinase-related protein 4 (DPYSL4)*	2	20	22	0	0	0	4.19	
	P07437	Tubulin beta chain (TUBB) *	0	0	0	3	168	125	-7.60	

† SC = Spectral count

* indicates protein that is absent in one group relative to the other

YA: Young Anterior vitreous; YP: Young Posterior vitreous; MA: Mature Anterior vitreous; MP: Mature Posterior vitreous

#### Mature anterior vitreous versus mature posterior vitreous

In the mature rabbits, 388 proteins were compared for abundance between anterior and posterior vitreous, and 11 proteins were found differentially expressed in anterior relative to posterior group (Table 1 “MA VS. MP”). Nine statistically more abundant proteins in the anterior compartment compared to the posterior compartment were actin cytoplasmic 1 (ACTB, p<0.001), elongation factor 1-alpha 2 (EEF1A2, p<0.05), CA3 (p<0.001), 14-3-3 protein zeta/delta (YWHAZ, p<0.001), CRYL1 (p<0.001), CRYAB (p<0.001), CRYAA (p<0.001), CRYBB2 (p<0.01) and CRYBB1 (p<0.05) as shown in Table 1 “MA VS. MP”. VIM and TUBB were statistically less abundant in the posterior compartment (both p<0.001). S1B Fig showed fold changes of these differentially expressed proteins between mature anterior and mature posterior vitreous. In addition, ACTB and EEF1A2 were not found in any samples of mature posterior vitreous.

#### Young anterior vitreous versus mature anterior vitreous

We compared the protein profiles of vitreous from the same region at different ages. Among 310 proteins identified in the anterior compartments, 4 proteins were differentially present between young and mature vitreous, including TUBB (p<0.001), YWHAZ (p<0.001), 3 alpha (17 beta)-hydroxysteroid dehydrogenase (PGER6, p<0.01) and ATPase, Na+/K+ transporting, alpha 3 polypeptide (ATP1A3, p = 0.01) as indicated in Table 1 “YA VS.MA”. S1C Fig showed fold changes of these proteins between the 2 groups. Further, YWHAZ, PGER6 and ATP1A3 were present only in mature anterior vitreous.

#### Young posterior vitreous versus mature posterior vitreous

Similarly, among 433 proteins detected in the posterior compartments, 4 proteins were statistically differentially expressed between young posterior and mature posterior vitreous, including TUBB2A (p<0.001), ACTB (p<0.001), DPYSL4 (p<0.01) and TUBB (p<0.001) (Table 1”YP VS. MP”). S1D Fig showed fold changes of these proteins. Further, TUBB2A, ACTB and DPYSL4 were detected only in young posterior vitreous while TUBB was detected exclusively in mature posterior vitreous.

### Confirmation of the differentially expressed proteins using WB

**Fig 3:**
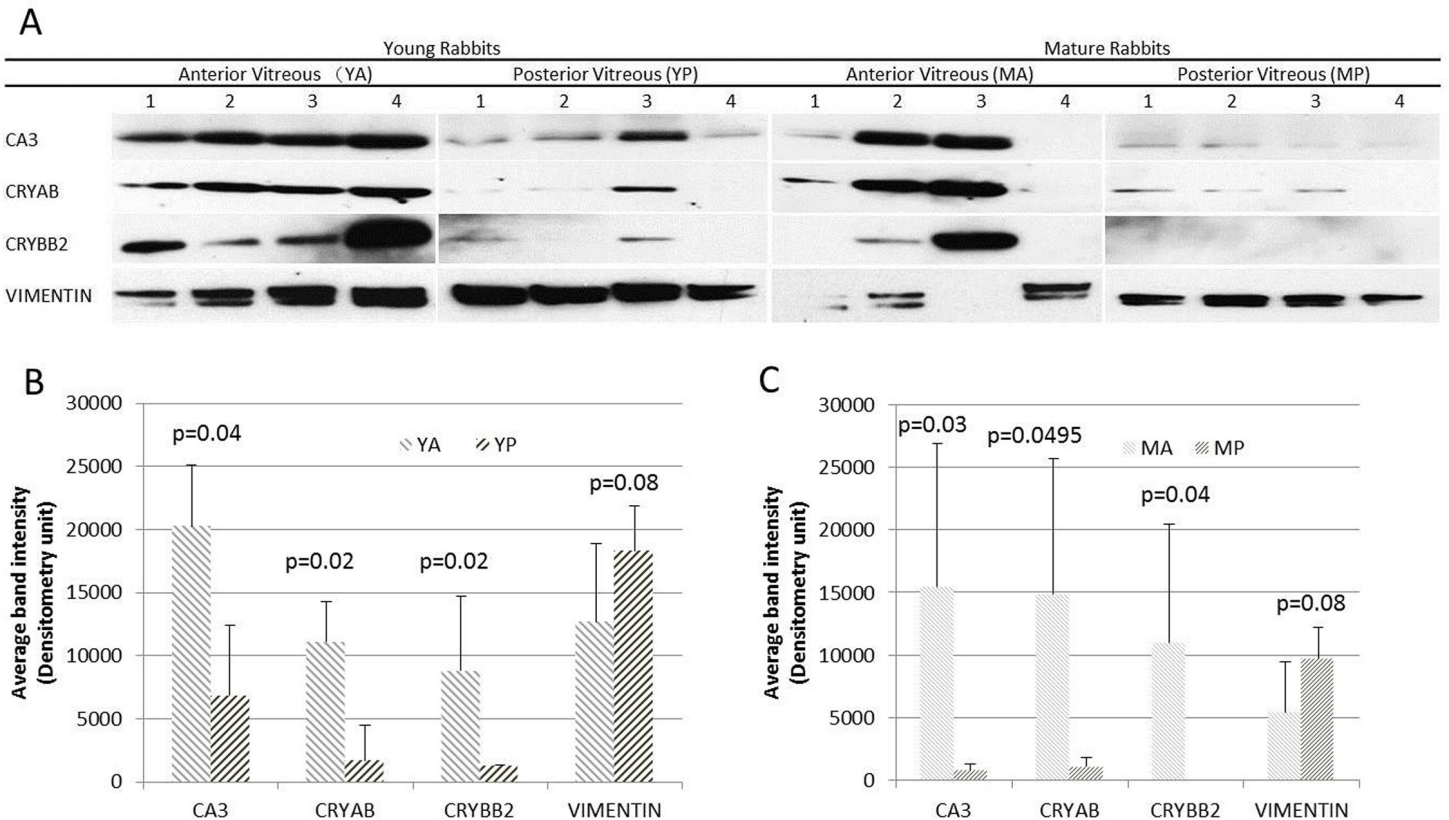
Western blot analysis of anterior and posterior vitreous of young and mature rabbits respectively using antibodies against (from top to bottom): CA3, CRYAB, CRY BB2 and vimentin (A) and Densitometric analysis of CA3, CRYAB, CRYBB2 and vimentin in young anterior vitreous (YA, downward diagonally striped bars) vs. young posterior vitreous (YP, upward diagonally striped bars) (B). (Values are listed as the mean ± SD). Densitometric analysis of CA3, CRYAB, CRYBB2 and vimentin in mature anterior vitreous (MA, downward diagonally striped bars) vs. mature posterior vitreous (MP, upward diagonally striped bars) (C).

WB analysis and densitometry of the blots (Fig 3) were used to confirm results obtained by LC-MS/MS. Similar to LC-MS/MS, CA3 was more abundant in young anterior vitreous than in young posterior vitreous (p = 0.04). The expression of CA3 in mature anterior vitreous was also higher than that in mature posterior vitreous (p = 0.03). CRYBB2 was more abundant in anterior compared to posterior vitreous in both young and mature rabbits (p = 0.02 and 0.04, respectively). The expression of CRYAB was also greater in the anterior vitreous in young and mature rabbits (p = 0.02 and 0.0495, respectively). However, there were no significant differences in the level of vimentin between young anterior and young posterior vitreous (p = 0.08) or mature anterior and mature posterior vitreous (p = 0.08). Although a visible difference was seen in the band thickness of vimentin between mature anterior and mature posterior vitreous in the WB, that difference was not statistically significant.

## Discussion

Previous studies have applied proteomics to normal vitreous humor using different separation and mass spectrometry techniques, and various numbers of proteins have been identified in the vitreous from human and mouse[4, 5, 18–27]. The total number identified in our study is relatively low in comparison to other similar studies. This is partially due to the different methodology used for LC-MS/MS such as sampling and lack of prefractionation. Some of those studies also included those proteins containing 1 matched peptide, leading to much greater number of proteins identified. In order to increase the confidence in the final panel of the differentially expressed proteins, we applied a relatively stringent statistical criteria and sorting methods described above. Using our method, we minimized the rate of false identification and compiled a list of proteins that were both significantly present in each sample and significantly different between age or region groups. To the best of our knowledge, none of the previous studies has identified the differentially abundant proteins between both different regions and ages. In this study, we detected 231 and 273 distinct proteins in the anterior vitreous of young and mature rabbits, respectively, and 375 and 353 proteins in the posterior vitreous of young and mature rabbits, respectively. The number of proteins detected in the posterior region was greater than of that detected in the anterior region in both young and mature rabbits. Additionally, there were more proteins shared between young posterior and mature posterior vitreous than between young anterior and mature anterior vitreous. This indicates that the protein profiles in the anterior and posterior vitreous are different. In young rabbits, 15 differentially abundant proteins were identified between the anterior and posterior regions with 11 more abundant in the anterior region and 4 more abundant in the posterior region. While in mature rabbits, there were 11 differentially abundant proteins with 9 more abundant in the anterior region and 2 more abundant in the posterior region. More proteins in the anterior region had greater abundance than in the posterior region. Comparing the protein profiles of the same region from rabbits at different ages, we found lesser differences in the abundance level. Among 310 proteins identified from young anterior and mature anterior vitreous, only 4 proteins were differentially abundant with either present only in young anterior or mature anterior vitreous. Similarly, 4 out of 433 proteins were differentially abundant between young posterior and mature posterior vitreous, and they were either detected in young posterior or mature posterior vitreous. Based on these data, it appears that differences in protein abundance are more pronounced between different regions than between the vitreous samples at different ages. In other words, age may have less influence on the abundance of proteins in vitreous humor than its anatomy.

Identification of crystallins in the vitreous and their confirmed differential abundance in regions of vitreous body is intriguing. Crystallins are proteins mainly found in the lens and the cornea of the eye as structural proteins accounting for the transparency of these structures[28]. One possibility that one might occur to a reader is the possibility of contamination of the vitreous sample from a damaged lens. However, the meticulous technique of vitreous sampling used ensured an intact lens in all the samples from which vitreous was harvested. In recent years, some studies revealed the existence of different types of crystallins in the vitreous and/or retina, suggesting the significance of crystallins as functional proteins that increase intracellular stability through their chaperone activity and possibly due to the interaction with cell signaling pathways[4, 26, 29–33]. The source of the crystallins in the vitreous is mainly from the cellular structures that come in contact with the vitreous, namely the ciliary epithelium, lens and retina. Our data showed that many crystallins were found more abundantly in the anterior than the posterior compartment. This difference may relate to the proximity of the anterior vitreous compartment to the crystalline lens, which potentially contributes to the higher crystallin content in the anterior compartment. It is also possible that these soluble proteins may enter the anterior vitreous from plasma by filtration through fenestrated capillaries of the ciliary body and the ciliary epithelium. Unlike young rabbits, the vitreous of mature rabbits showed gamma-crystallin and beta crystallin A1, B3 levels were similar in the anterior and posterior compartments, while CRYL1, CRYAB, CRYAA, CRYBB1 and CRYBB2 levels still remained higher in the anterior compartment of mature rabbits as they did in young rabbits. This suggests that the region-related abundance difference of crystallins may or may not change with age. Makoto Kodama et al. revealed that the vitreous liquefaction is induced by detachment of non-collagenous protein beads containing crystallins, resulting in the collapse of the structure to release watery liquid trapped within the meshwork[34]. It remains unknown whether the changes of certain crystallins in the anterior compartment with age play a role in liquefaction. Meanwhile, it is becoming increasingly clear that crystallins have important metabolic and regulatory functions, both within the lens and in other parts of the eye including the vitreous. Further research is warranted to investigate the differential abundance of these crystallins and their potential roles in the vitreous humor.

Ocular carbonic anhydrases (CAs) received much attention for their important role in aqueous humor production and regulating intraocular pressure [35]. By using histochemical staining, prior studies identified the soluble isozymes CA1 in cornea, lens, ciliary process and choroid; CA2 in cornea, lens, ciliary process and retina; and CA4 in ciliary body and lens; but not CA3 in retina, iris, lens or ciliary body [35, 36]. Recently Skeie and Mahajan detected CA3 in mouse vitreous through LC-MS/MS, and their proteomics data showed greater CA3 expression in vitreous than retina, which indicates that CA3 in vitreous may not originate from retina[26]. Our data showed that CA3 level was higher in the anterior compartment in both young and mature rabbits, suggesting that CA3 may be mainly secreted from the ciliary body into the vitreous humor. Further investigation on whether CA3 has a physiological function in the vitreous humor seems worthwhile.

ALDH1A1 was more abundant in young anterior than young posterior vitreous, but was detected at similar levels between mature anterior and mature posterior vitreous. The aldehyde dehydrogenase (ALDH) family of proteins consists of cytosolic isoenzymes responsible for oxidizing intracellular aldehydes, thus contributing to the oxidation of retinol to retinoic acid in early stem cell differentiation[37]. Class 1 of the ALDH family (ALDH1) is the isoform of ALDH that predominates in mammals. We speculate the relative different detection levels in ALDH1 between regions could be due to aging.

We also analyzed the proteins identified in only one group relative to the other group with statistical significance. In young rabbits, TUBB was found only in the anterior compartment, while DPYSL4 and TUBB2A were detected only in the posterior compartment. In mature rabbits, EEF1A2 and ACTB were identified only in the anterior compartment. In anterior vitreous, 2 proteins (ATP1A3 and PGER6) were exclusively detected in mature rabbits; while in the posterior vitreous, 3 proteins (DPYSL4, ACTB and TUBB2A) were identified exclusively in young rabbits, TUBB exclusively in mature rabbits. These proteins could potentially be age or region specific. Furthermore, DPYSL4 and TUBB2A were detected only in young posterior vitreous, while PGER6 was found only in mature anterior vitreous but not in other 3 groups. These 3 proteins could be both age and region specific. DPYSL4, a known regulator of hippocampal neuron development, has important roles in dendrite arborization, guides post navigation and neuronal plasticity and has been shown to be involved in neural polarity and differentiation in mice[38]. PGER6, also known as prostaglandin-E(2)9-reductase, is a monomeric cytoplasmic enzyme that catalyzes reduction of many carbonyl compounds. These proteins are expressed exclusively in certain regions or at certain ages. Their unique presence or absence may be associated with age or region related biochemical pathway, and further investigations are required.

One potential weakness of this study is the relatively small number of rabbits studied. However, the consistency of the protein profile among the samples and the significant differences in the different groups suggested that the number of animals may not have had a significant effect. It is possible that certain proteins that are minute in quantity may have shown significant differences if a larger sample size was used. Furthermore, it is possible that in older rabbits, there might be additional changes in the vitreous protein profile. However, such animals were not available for study from approved vendors. Therefore, the findings in this study should not be interpreted as aging change but as demonstration that the vitreous protein profile is dynamic and that changes occur between young rabbits and ones that have reached sexual maturity at six months[13]. In addition, because of the large number of the new proteins identified in the rabbit vitreous that were not previously reported in human or rodent vitreous, it is possible that the rabbit may have a different protein profile compared to humans and rodents, therefore, caution should be taken in interpreting data from different species. It is also possible that sample preparation techniques used in this and other studies were different and may have resulted in the protein differences seen between our study and those previously published[18–21]. Additional comparative protein profiling of the rabbit vitreous and the vitreous protein profile of human and mouse using the same sample preparation and analysis methods might be wanted.

In summary, our study revealed differences in the constitution of the vitreous proteome between young and mature rabbits as well as between anterior and posterior compartments. We speculate such compositional differences may result from the different surrounding tissues, which secrete or shed different proteins at different ages, and the viscosity of the vitreous, which may confine exchange of proteins between anterior and posterior compartments. The age or region related differences in the detection level of proteins may provide useful information for vitreous research, help establish baseline protein profiles of vitreous in different regions at different ages, and develop disease biomarkers with respect to the age and location.

## Supporting Information

S1 Fig Bar Graphs showing the fold changes (log ratio) of the differentially expressed proteins in young anterior vitreous (YA) relative to young posterior vitreous (YP) (A), mature anterior vitreous (MA) relative to mature posterior vitreous (MP) (B), YA relative to MA (C) and YP relative to MP (D) based on LC-MS/MS data. Ratios were calculated by dividing spectral counts in one group/ spectral counts in other group.
(TIF)

S1 Table Table A in S1 Table: A full list of identified proteins in our study; Table B in S1 Table: A list of proteins identified in our study but not previously reported. (XLSX)

S2 Table Table A,B,C and D in S2 Table: A comprehensive list of proteins identified in young anterior group, young posterior group, mature anterior group and mature posterior group respectively. (XLSX)
