# Supplementary figures and images for: Differential Expression of Vitreous Proteins in Young and Mature New Zealand White Rabbits
Source: PLoS One. 2016 Apr 18;11(4):e0153560. doi: 10.1371/journal.pone.0153560 (PMC4835093; doi:10.1371/journal.pone.0153560)

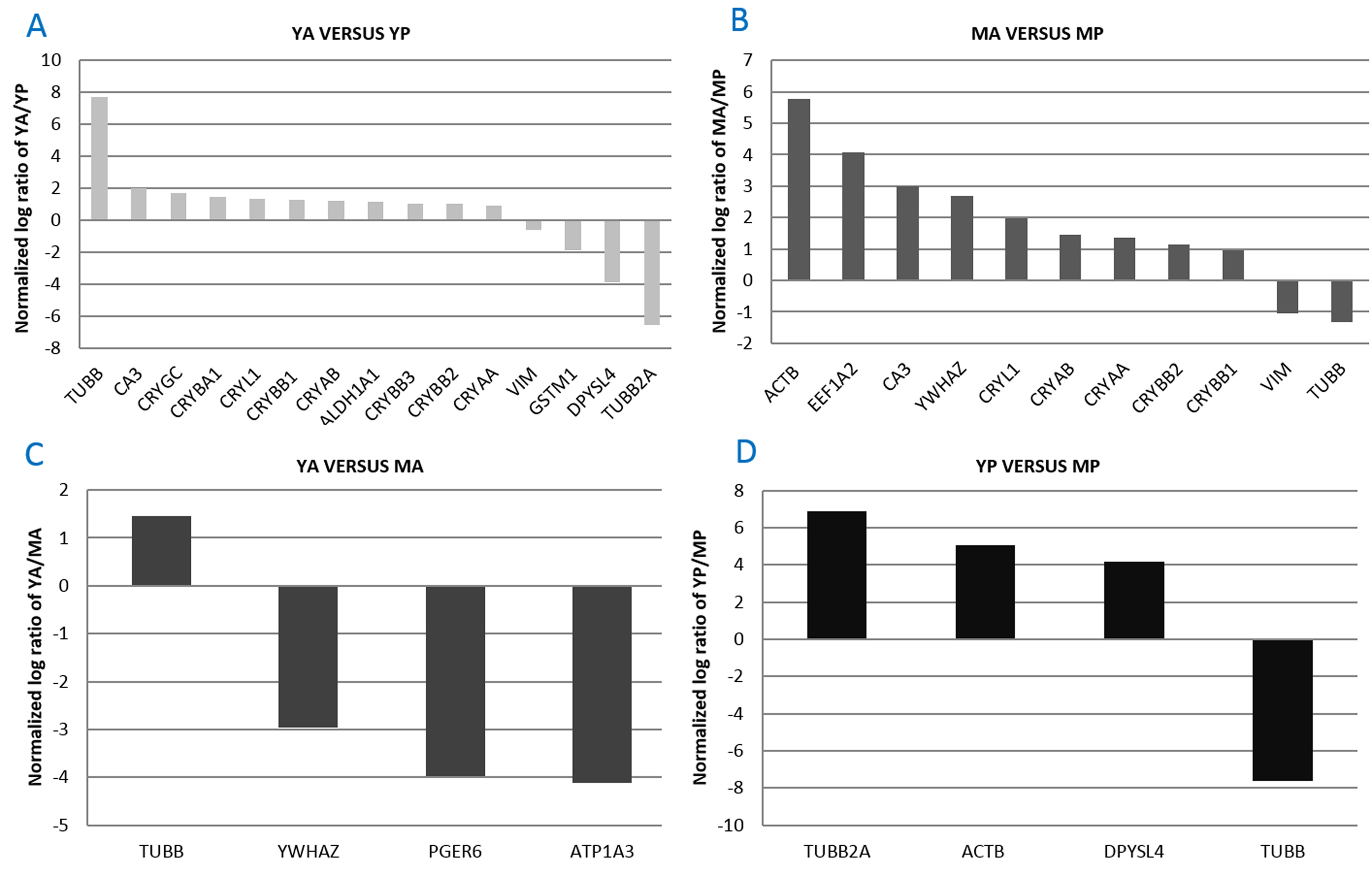

Supplement: S1 Fig — Bar Graphs showing the fold changes (log ratio) of the differentially expressed proteins in young anterior vitreous (YA) relative to young posterior vitreous (YP) (A), mature anterior vitreous (MA) relative to mature posterior vitreous (MP) (B), YA relative to MA (C) and YP relative to MP (D) based on LC-MS/MS data. Ratios were calculated by dividing spectral counts in one group/ spectral counts in other group. (TIF) [file pone.0153560.s001.tif]
